# Legitimacy and Implications of Reducing *Colletotrichum kahawae* to Subspecies in Plant Pathology

**DOI:** 10.3389/fpls.2016.02051

**Published:** 2017-01-09

**Authors:** Dora Batista, Diogo N. Silva, Ana Vieira, Ana Cabral, Ana S. Pires, Andreia Loureiro, Leonor Guerra-Guimarães, Ana P. Pereira, Helena Azinheira, Pedro Talhinhas, Maria do Céu Silva, Vitor Várzea

**Affiliations:** ^1^Centro de Investigação das Ferrugens do Cafeeiro, Instituto Superior de Agronomia, Universidade de LisboaOeiras, Portugal; ^2^Linking Landscape, Environment, Agriculture and Food, Instituto Superior de Agronomia, Universidade de LisboaLisboa, Portugal; ^3^Computational Biology and Population Genomics Group, Centre for Ecology, Evolution and Environmental Changes, Faculdade de Ciências, Universidade de LisboaLisboa, Portugal

**Keywords:** coffee berry disease, anthracnose, *Colletotrichum* taxonomy, *Colletotrichum gloeosporioides* species complex, *Colletotrichum kahawae* subsp. *kahawae*, *Colletotrichum kahawae* subsp. *ciggaro*

*Colletotrichum kahawae* Waller and Bridge is a highly aggressive and specialized fungal pathogen of coffee, causing the devastating Coffee Berry Disease (CBD), particularly at high altitudes. The disease arises from the unique ability of the pathogen to infect green developing coffee berries. This pathogen is currently confined to the African continent in all countries that grow Arabica coffee (*Coffea arabica* L.), leading to up to 80% yield losses, if no control measures are applied (Silva et al., 2006; Vossen and Walyaro, 2009; Hindorf and Omondi, 2011). For such huge economic impact, it is ranked as a quarantine pathogen and even as a biological weapon (Australia Group, 2014). Consequently, the pathogen's potential dispersal to other Arabica coffee cultivation regions is greatly feared, particularly to those at high altitude also found in Latin America and Asia. Recently, this recognized species was brought down to a subspecific level (*C. kahawa*e subsp. *kahawae*) based on molecular data (Weir et al., 2012), clustering together with a generalist and cosmopolitan group of *Colletotrichum* isolates unable to cause CBD (*C. kahawae* subsp. *ciggaro*). Since then a growing number of studies have reported the identification of *C. kahawae* in various hosts and regions of the world (Liu et al., 2013; Afanador-Kafuri et al., 2014; Mosca et al., 2014; Schena et al., 2014; Ismail et al., 2015; Garibaldi et al., 2016a,b; Perrone et al., 2016). Although these reports are referring to *C. kahawae* subsp. *ciggaro*, some of them could not distinguish the pathogen at the subspecific level, and this is leading to a wave of confusion of whether the long accepted species *C. kahawae*, the CBD pathogen, has escaped from Africa and extended its host range. Given the extreme impact that this situation may trigger and the subsequent biosecurity implications, there is a practical need to completely distinguish these pathogens taxonomically as to avoid the risk of misidentification, and caution should be taken on assigning/reassigning taxonomic ranking and nomenclature. Here we consider the evidences sustaining and contradicting the classification proposed by Weir et al. (2012), and discuss the risks and practical implications of changing the CBD pathogen's species status in a plant pathology context.

After a long period of confusion in nomenclature, *Colletotrichum kahawae* (epithet derived from the Swahili word for coffee) was proposed by Waller et al. (1993) as a formal name to refer specifically to *Colletotrichum* isolates causing CBD and taxonomically distinguishing them from other *Colletotrichum* strains found on coffee. The CBD pathogen was previously known as either *C. coffeanum* var. *virulans* (Rayner, 1952), a form of *C. coffeanum* (Nutman and Roberts, 1960), CBD-strain (Gibbs, 1969), or *C. coffeanum* Noack sensu Hindorf (Hindorf, 1970, 1974). Interestingly, the name *C. coffeanum* was not based on type material associated with CBD, but on samples from Brazil where the disease does not exist, and it clearly referred to *C. gloeosporioides* (Waller et al., 1993), thus in view of its distinctive characteristics the applicability of these names was questionable. Waller et al. (1993) argued that, although closely related with *C. gloeosporioides*, the CBD pathogen is sufficiently distinct in pathogenicity and related ecology, morphological, cultural, and biochemical characteristics to warrant recognition as a distinct species. This was further confirmed based on multi-locus phylogenies (Prihastuti et al., 2009; Silva et al., 2012a).

Recently, an alteration of the species taxonomic status of *C. kahawae* was proposed by Weir et al. (2012), placing the CBD pathogen again in an elusive position. Using a multi-locus sequencing approach, both Silva et al. (2012b) and Weir et al. (2012) showed that the CBD pathogen share a remarkable genetic similarity with a group of isolates found on a wide range of hosts and geographical origins [referred as Undescribed Group 1 (UG1) by Silva et al., 2012b]. Indeed, for a set of barcoding gene sequences (*ACT, CAL, CHS-1, GAPDH, TUB2, SOD2*, and *ITS*) the two taxa are indistinguishable. In their revision of the *C. gloeosporioides* species complex, Weir et al. (2012) accepted species according to the genealogical concordance phylogenetic species recognition (Taylor et al., 2000) criteria alone, and as such both groups should indeed be regarded as a single species. Thus, standing solely from a phylogenetic point of view, Weir et al. (2012) recognized them as two subspecies: the CBD pathogen (*C. kahawa*e subsp. *kahawae*) and the genetic similar *Colletotrichum* isolates (*C. kahawae* subsp. *ciggaro*; as a side note to the nomenclature chosen by the authors, the Portuguese word for cigarette is *cigarro*). However, as further demonstrated by Silva et al. (2012b) through population genetic, evolutionary, and pathological data, these clearly represent ecologically distinct and reproductively isolated biological entities, which have separated quite recently. According to the authors, UG1 (*C. kahawae* subsp. *ciggaro sensu* Weir et al., 2012) seems to be a sibling lineage from which *C. kahawae* may have undergone a very recent ecological speciation through host jump into *C. arabica*, and thus still share a considerable genetic background, although have become quite differentiated, as shown by the significant and elevated differentiation indexes across all studied loci and a complete segregation of polymorphic sites (Silva et al., 2012b). Its unparalleled adaptation of infecting green coffee berries enables the CBD pathogen to occupy a unique ecological niche, which separates it on a functional level from all other *Colletotrichum* species (Figure 1). Quite a list of distinctive features can follow. In addition to its biology and slow growth form, the CBD pathogen can be distinguished metabolically by its inability to use either citrate or tartrate as a sole carbon source (Waller et al., 1993 and references herein; Varzea et al., 2002; Weir et al., 2012). At the genetic level, single genes or a combination of genes, specifically glutamine synthetase (*GS*) (Weir et al., 2012), mating type 1-2-1 (*MAT1-2-1*), and a fragment of DNA lyase *Apn*2 (*Apn*25L) (Silva et al., 2012b), reliably distinguish the CBD pathogen, as well as the genome size (Pires et al., 2016).

**Figure 1:**
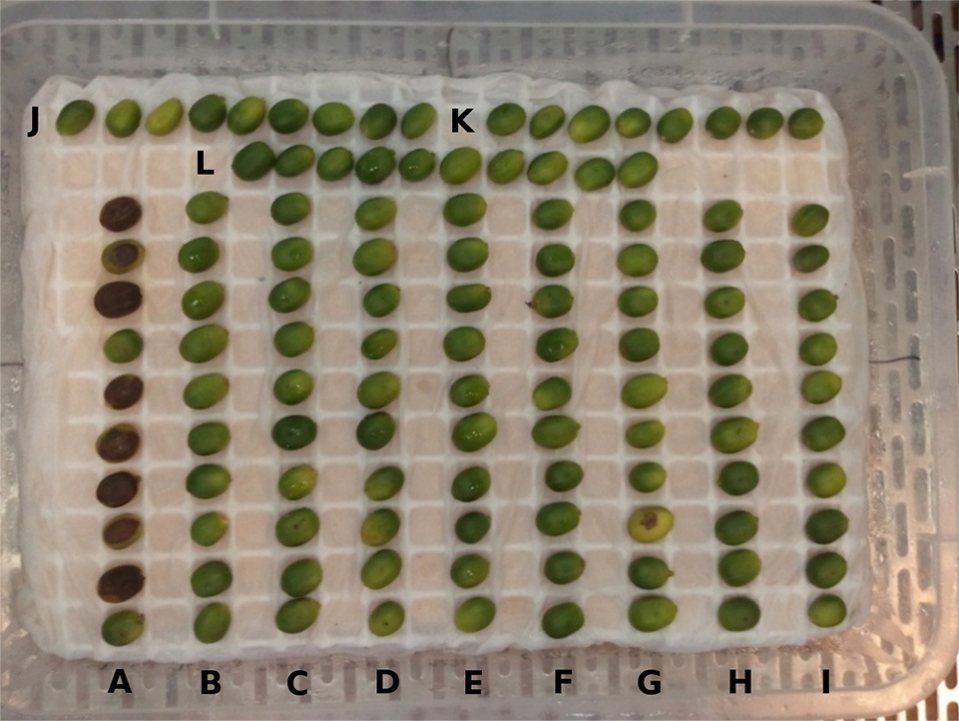
**Pathogenicity tests on coffee green berries showing that only ***C. kahawae*** subsp. ***kahawae*** isolates produced necrotic and sunken lesions characteristic of coffee berry disease symptoms (isolate Cam1, A),** when compared to isolates from *C. kahawae* subsp. *ciggaro* [ICMP 17922 **(C)**; ICMP 18534 **(E)**; ICMP 12953 **(F)**; ICMP 18539 **(G)**] and from other close phylogenetic groups [Cg432 **(B)**; *Glomerella cingulata* “f. sp. *camelliae*” ICMP 18542 **(D)**; *C. aotearoa*, ICMP 18530 **(H)**, ICMP 18537 **(I)**, ICMP 18541 **(J)**, ICMP 18536 **(K)**, ICMP 17326 **(L)**]. Isolates (**C–L)** were kindly provided by Bevan Weir and Peter Johnston (Landcare Research, Auckland, New Zealand), and Cg432 by Ana Paula Ramos (Instituto Superior de Agronomia, Universidade de Lisboa, Lisboa, Portugal).

In the light of all these evidences reinforcing the differentiation of the CBD pathogen as a distinct and quite separate taxon in the coffee mycobiota, the taxonomic treatment proposed by Weir et al. (2012) is debatable and should be carefully interpreted. The authors acknowledge its distinctive features and biosecurity importance and to reflect this particularity retain a distinct taxonomic label for the CBD pathogen, while choosing to accept it only as biologically specialized population within a monophyletic supported species. It is well known that the species concepts are still in a state of flux in *Colletotrichum* taxonomy (Crouch et al., 2014), and particularly the low levels of genetic divergence across the *C. gloeosporioides* complex provide a technical challenge for species resolution (Silva et al., 2012a). Even more so, Weir et al. (2012) recognize the continuing uncertainty about sensible species limits within the *C. gloeosporioides* complex and that more powerful genes than the limited set sampled in their study, may provide finer resolution within the species-level clades that they accept. Precisely for this reason, extreme caution should be taken when assigning taxonomic ranking within members of the *C. gloesporioides* complex and additional data should be consider to complement genetic diversity and phylogenetic information, especially when biosecurity issues are at stake. In this case, the name *Colletotrichum kahawae* has a strong practical meaning and an indissoluble connection with the coffee disease. Bringing a volatile group of fungi able to infect a wide range of organs and hosts worldwide under the same specific epithet (which means coffee), is likely to disrupt the accuracy required in the nomenclature for pathogen identification and cause a great deal of confusion, even if distinction at the subspecific level is provided.

As a predictable consequence, several disease notes have reported the identification of *C. kahawae* in various hosts and regions out of Africa, including *Rubus glaucus* in Colombia (Afanador-Kafuri et al., 2014), olive, mango, and mandarin in Italy (Mosca et al., 2014; Schena et al., 2014; Ismail et al., 2015; Perrone et al., 2016), *Banksia* sp. in Portugal (Madeira) and Spain, and *Leucospermum* sp. in the USA (Hawaii) (Liu et al., 2013). While these studies assigned their isolates to *C. kahawae* subsp. *ciggaro*, others did not applied the genetic markers able to distinguish the two subspecies, and dangerously reported the presence of *C. kahawae* (no subspecies given) on rocket (*Eruca sativa*) (Garibaldi et al., 2016a) and American sweetgum (*Liquidambar stryraciflua*) (Garibaldi et al., 2016b) in Italy, suggesting the adoption of measures to contain the spread of the pathogen. The current classification promotes the chance of misidentification and uncertainty of the taxon addressed, leading to inaccurate records, distress within the coffee research and technical community, and severe biosecurity implications. On one hand, this might provoke a situation of assuming that *C. kahawae* has become able of infecting other hosts, and as such, of causing a devastating disease similar to that in coffee. On the other, *C. kahawae* is listed under strict quarantine regulations in many coffee growing countries, such as Australia and China (Jayawardena et al., 2016), and upon the information of the pathogen's potential detection, a state of emergency could be declared, particularly in major producing countries which have their economy strongly based on coffee production. Accurate naming of *Colletotrichum* to species level is crucial with regard to species identification and diversity, plant pathology and quarantine, agriculture, bio-control, plant breeding, whole genome sequencing, developing, and maintaining knowledge data bases, commercial applications and understanding evolutionary history (Jayawardena et al., 2016). If cryptic species are mistaken for a single species, then the species' integrity and understanding is compromised. Whether the *C. kahawae* subspecies' recognized by Weir et al. (2012) are after all genetically good species, will most likely be only suitably resolved with genomic data, an endeavor that is close to be accomplished (Vieira et al., 2016). For now, it should be considered that, as shown by Silva et al. (2012b) and Pires et al. (2016), notwithstanding the remarkable genetic similarity, there are enough evidences to accept them as distinct species, and in this light they would be better described and viewed as cryptic species.

## Author contributions

DB, MCS, and VV conceived the idea. DB, DNS, AV, AC, ASP, AL, LGG, APP, HA, PT, MCS, and VV contributed to develop the concept and structure of the manuscript. DB wrote the manuscript. DNS prepared and edited the figure. DNS, AV, AL, LGG, HA, PT, MCS, and VV provided revisions to the manuscript. All authors read and approved the work for publication. The views and opinion presented in this article are those of the authors.

### Conflict of interest statement

The authors declare that the research was conducted in the absence of any commercial or financial relationships that could be construed as a potential conflict of interest.
